# Concomitant vagal and adrenergic stimulation does not precipitate ventricular arrhythmias in a healthy rabbit heart model of autonomic conflict

**DOI:** 10.1186/2046-7648-4-S1-A39

**Published:** 2015-09-14

**Authors:** James Winter, Michael J Tipton, Michael Shattock

**Affiliations:** 1 Cardiovascular Division, King's College London, UK; 2 Extreme Environments Laboratory, DSES, University of Portsmouth, UK

## Introduction

Experimental studies indicate that cold-water immersion is associated with incidence of supraventricular and ventricular arrhythmia in young, seemingly, healthy subjects [1]. We recently hypothesised that the sudden and simultaneous sympathetic and parasympathetic activity, termed "autonomic conflict" (AC), may be a cause of arrhythmias associated with cold-water immersion and, when combined with other risk factors, may explain immersion-related sudden death. The aim of the present study was to investigate the arrhythmic consequences of AC in normal healthy rabbit hearts.

## Methods

Adult New Zealand White rabbits (n = 9) were anaesthetised and their hearts isolated with intact autonomic innervation. Isolated preparations were perfused in constant pressure (80 mmHg) in the Langendorff mode and instrumented for electrocardiogram recordings. The effects of right vagus nerve stimulation (VNS - 10 Hz, 5 V) were assessed in control conditions and during perfusion with noradrenaline (100 nmol.L^-1^) and adrenaline (25 nmol.L^-1^) (CAT). Data represent mean(SEM). *P < 0.05.

## Results

**Figure 1:**
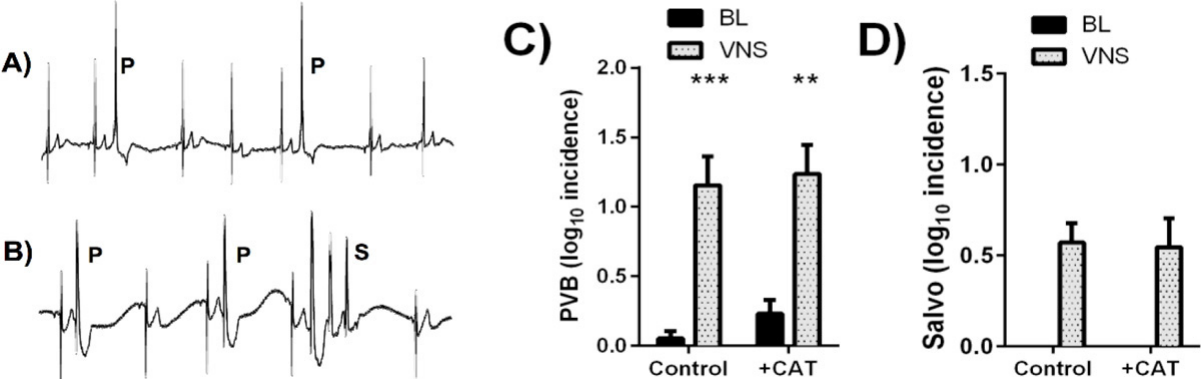
**Ventricular arrhythmias in the isolated rabbit heart during vagal nerve stimulation (VNS) with and without background catecholamine perfusion (+CAT)**. BL=baseline, P=premature ventricular beat (PVB), S=ventricular salvo (n = 9)

VNS was associated with rapid onset bradycardia that was potentiated by CAT perfusion (Δ heart rate = -80(6) vs. -122(8) bpm, P < 0.01). As shown in Figure 1, VNS alone was associated with spontaneous ventricular arrhythmias, including premature ventricular beats (PVBs), ventricular salvos, and sustained atrio-ventricular dissociation (not shown). However, when VNS was combined with CAT there was no further increase in either PVBs (C) or salvos (D).

## Discussion

The results of the present study indicate that AC does not precipitate ventricular arrhythmia in the healthy rabbit heart. Rather we observe that PVBs can arise spontaneously as a consequence of a strong vagal stimulus. The mechanism of arrhythmias associated with VNS is not immediately apparent, however the prematurity of ventricular beats, in most cases, suggests that these beats are not likely to be escape rhythms attributable to cardiac slowing, but rather true extrasystoles.

## Conclusion

In the absence of co-morbidities, we find no evidence in the healthy rabbit heart in support of the AC hypothesis as the cause of arrhythmic episodes associated with cold-water immersion. The effect of superimposition of AC on other risks factors, such as cardiovascular disease, has yet to be investigated.
